# Microwave Properties of One-dimensional Photonic Structures Based on Composite Layers Filled with Nanocarbon

**DOI:** 10.1186/s11671-017-2034-8

**Published:** 2017-04-13

**Authors:** Ludmila Vovchenko, Oleg Lozitsky, Igor Sagalianov, Ludmila Matzui, Vilen Launets

**Affiliations:** Department of Physics, Taras Shevchenko National University of Kyiv, Volodymyrska str., 64/13, Kyiv, 01601 Ukraine

**Keywords:** Photonic crystal, Composite layer, Graphite nanoplatelets, Carbon nanotubes, Dielectric permittivity, Electromagnetic radiation, Transmission index

## Abstract

This work presents the results of computer modeling and experimental measurements of microwave transmission properties for one-dimensional periodic multi-layered photonic structures (PCs), composed of epoxy layers and composite layers filled with nanocarbon particles—multi-walled carbon nanotubes and graphite nanoplatelets. The results show that the characteristics of observed photonic band gaps in transmission spectra of PC can be controlled by varying the parameters of layers, namely, the complex permittivity and the layer thickness. It was found that the insertion of the defects (for instance, magnetic layer) into photonic structure can change the EMR transmission spectrum. The comparative analysis of EMR transmission spectra for investigated photonic structures has showed good agreement between the experimental and simulated data. It was found that EMR absorption in composite layers of photonic structures shifts the transmission spectra to the smaller values of EMR transmission index and reduces the sharpness of photonic band gaps. Thus, by changing the parameters of composite layers in photonic structure, we can obtain the tunable photonic band gaps, necessary for technological applications in devices, capable of storing, guiding, and filtering microwaves.

## Background

Photonic crystals (PCs) attract large attention due to their wide application ability. PCs are macroscopic media which arranged periodically with different refractive indices, and their periodicities are in the range of the incident light [1]. Photonic structures (PS) with photonic band gap (PBG) allow propagation of electromagnetic radiation (EMR) only in certain frequency bands, while the incident radiation for other frequency bands is almost completely reflected [1, 2]. This property is important for various applications of PC, including the manipulation of optical radiation in laser devices and information transmission systems [3]. The functionality of PC can be significantly enlarged by controlling their spectral properties by varying geometrical or internal parameters of the structure. Most important property of PC is periodic distribution of areas with high contrast of electromagnetic properties. In such structures, the permittivity is a periodic function in space. In case when the dielectric permittivity function repeats itself in one dimension (1D) the structure is called one-dimensional photonic crystal (1D PC) [4], if it repeats itself in 2D or 3D the structure is called 2D or 3D PC [5, 6].

The simplest form of PC is one-dimensional periodic structure, which consists of packet of layers, which have low and high refraction indices (related with low and high dielectric permittivity, ${\varepsilon}_{r1}^{\prime }$ for L layer and ${\varepsilon}_{r2}^{\prime }$ for H layer) are located sequentially. When layers thicknesses *d*_1_ and *d*_2_ satisfy the Bragg condition $\sqrt{\varepsilon_{r1}^{\prime }}\cdot {d}_1={\lambda}_0/4$, $\sqrt{\varepsilon_{r2}^{\prime }}\cdot {d}_2={\lambda}_0/4$, a single photonic band gap is observed with center at the reference frequency *f*_0_ = *C*_0_/*λ*_0_, where *C*_0_ = 3 ⋅ 10^8^ m/s.

The propagation of photons in the PCs is similar to the propagation of electrons in the semiconductor crystals, where the effect of the periodic dielectric function on the propagating photon in PCs is much like the effect of the periodic potential function on the propagating electron in semiconductor crystal. Consequently, a photonic band is created in PCs is similar to the electronic band gap in semi-conductor crystal [3]. As electronic band gaps represent the main feature behind all semi-conductor devices, photonic band gap (PBG) structures could also provide means for similar control of light [7, 8]. Regarding the materials for PBG structures, the periodic modulation of the dielectric constant can be achieved by periodically structuring a dielectric. Also, periodic metallic or metal-dielectric structures can be used [9, 10].

Although one-dimensional photonic crystals (1D PCs) are the simplest among these types due to the ease of fabrication and analyzation, they are interesting due to their numerous promising applications. For example, these 1D PC structures are the basis for creation of photonic filters operating over a wide frequency range, from radio waves up to optical wavelengths. According to the application type and the desired specifications, the required photonic filter differs among wide band, narrow band, or selective pass/stop filters at selected wavelength ranges. The problem of the finding the optimal design of 1D PC that best fits the desired performance is considered in some recent publications [11–14].

Photonic spectrum of the structure can be modified by violating its periodicity causing the band gap to have a mini-zone of transmission which corresponds to “defect” localized mode [15–17]. Recently, not only periodic structures of PC with defects are important in terms of physical properties but also quasiperiodic systems (quasicrystals) [18]. Among such quasiperiodic structures, there are distinguished two known quasiperiodic sequences: generalized Fibonacci sequence [19–21] and generalized Thue-Morse sequence [22, 23]. Quasiperiodic multi-layer systems can be considered as corresponding models for describing transition from the ideal periodic structures to random structures [24]. Quasiperiodic structures have a set of interesting physical properties, such as existence of PBG for various frequency ranges, which have found wide application of these structures as optical frequency-selective filters, in devices, meant to store, control, and filter electromagnetic radiation. The use of dielectric layers with the large difference in the value of permittivity or dielectrics with periodically incorporated metal rods/plates as elements of PC substantially restricts the possibility of adjusting the characteristics of the PBG and passbands, since there is a limited set of dielectrics with a discrete set of high values of permittivity and low dielectric loss in the microwave range, as well as metals with a discrete set of the values of conductivity. Therefore, it is promising to use the polymer composite materials at the designing of PCs, since the dielectric properties of composite layers can be varied within the wide range depending on the type and concentration of filler in composite [25]. In addition, the polymers filled with comparatively low content of carbon or dielectric particles are corrosion-stable, light, easily formable, and cheaper compared to traditional materials. So, the nanocomposites with fillers based on BaTiO_3_ and TiO_2_ [26, 27] showed high permittivity values $\left({\varepsilon}_r^{\prime }=10-30\right)$ and relatively low dielectric loss in the frequency range 8–18 GHz. Sufficiently high dielectric constant ${\varepsilon}_r^{\prime }$ was observed for polymer CM filled with nanocarbon fillers, carbon nanotubes (CNTs), and graphite nanoplatelets (GNPs) [28–30] at relatively low filler content due to high aspect ratio of nanocarbon particles and large contribution of interfacial polarization. As it was shown in our previous papers [31–33], the permittivity ${\varepsilon}_r^{\prime }$ of epoxy composites filled with GNPs and CNTs may range within 4–50 depending on the filler content and slightly decreases with the frequency in the range 26–54 GHz. Dielectric loss tangent $\tan \delta ={\varepsilon}_r^{{\prime\prime} }/{\varepsilon}_r^{\prime }$ for such CMs is 0.02–0.3, which unfortunately will lead to distortion of photonic band gaps in PCs, and today, only few papers are presented in the literature concerning the study of the impact of the electromagnetic losses in layers of PCs on EMR reflection and transmission spectra [34]. Another disadvantage of these composites is that a significant increase in the dielectric permittivity ${\varepsilon}_r^{\prime }$ of these composites with increasing content of highly anisometric nanocarbon conductive particles occurs in a narrow concentration range, limited by low percolation threshold for such systems, since after percolation, the transition to through-conduction and substantial electric losses occur.

Recently, some ways to increase the content of isolated anisotropic conductive filler particles, which act as “artificial dipoles” and significantly increase the permittivity of the CMs have been proposed. So, along with the carbon filler, the use of second dielectric filler will prevent the formation of conductive carbon network [35, 36], and on the other hand, the additional increase of interfacial regions enhances the role of interfacial polarization in the formation of effective permittivity of CM. Another way to increase permittivity of CMs consists in formation of insulating polymer layer on the surface of conductive filler before introducing them into the polymer matrix at CMs fabrication [37]. The using of hybrid conductive filler with a special particle morphology such as GNP with synthesized on their surface CNTs also leads to enhancement of CMs permittivity [38]. The using of composite layers as elements of 1D PC open new perspectives due to possible tuning of spatial distribution of anisometric filler particles in the layer during the manufacturing process by the action of the external fields [39]. So, the alignment of nanocarbon fillers may lead to anisotropic permittivity of composite and EMR transmission spectra for such PCs are strongly dependent on the polarization of EMR. Moreover, as it is shown in [40], the optical properties of the obtained composite 1D photonic crystals based on Si and liquid crystal (LC) can be tuned by means of electro- and thermo-optical effects. And finally, for the creation of a narrow tunable passband in PCs, a composite magnetic layer as defective layer can be used, with varied magnetic permeability and magnetic loss dependent on magnetic filler content.

The aim of this paper is to investigate 1D photonic structures performing the computer modeling and experimental measurements of EMR transmission index that allow us to fabricate the PSs with regulated number, depth, and width of photonic band gaps. The modeling of frequency dependences of transmission indexes has been performed for PS, placed in rectangular waveguide for frequency ranges of 26–37.5GHz and 37.5–54 GHz. In order to determine the possibility of use for designing PC, the composite layers filled with anisometric conductive particles as well as to determine the effect of the EMR absorption in such composite layers on microwave transmission spectra, and the parameters of the band gap in PC the periodic composite multi-layer structures with various composite layers filled with nanocarbon particles were fabricated and were investigated in the frequency ranges of 26–37.5 and 37.5–54 GHz. We also present the comparative analysis of the experimental and the calculated frequency spectra of EMR transmission index for prepared PCs.

## Methods

1D PS were fabricated on the basis of epoxy resin Larite (L285) and epoxy composite layers filled with nanocarbon particles–graphite nanoplatelets (GNPs) and carbon nanotubes (CNTs). The prepared PSs were composed of five layers of L285 (L-layers) which alter with composite layers (H-layers) based on L285, filled with 2 wt.%CNT (PS1), or with composite layers 5 wt.%GNP/L285 (PS2, PS3). Graphite nanoplatelets (diameter 0.2–30 μm, thickness 5–65 nm) were prepared according to a scheme described in [41]. Multi-walled carbon nanotubes were purchased from Cheap Tubes Inc. (purity ~90%, outer diameter 10–30 nm, length 10–30 μm). The parameters of fabricated photonic structures are presented in Table 1.

**Table 1:** Parameters of fabricated photonic structures based on epoxy L-layers and composite H-layers

Specimen number	Thickness of epoxy L-layer	Composition of composite H-layer	Thickness of composite H-layer
	*d*_1_, mm		*d*_2_, mm
PS1	5.0	2 wt.%CNT/L285	1.00
PS2	4.8	5 wt.%GNP/L285	0.48
PS3	4.3	5 wt.%GNP/L285	0.73
PS4	4.8	5 wt.%GNP/L285(4 layers)	0.48 (H-layer)
		+30 wt.%BaM(1 layer)	0.50 (M-layer)

For the epoxy L-layer real part of permittivity ${\varepsilon}_r^{\prime }$ is 2.9, while for composite H-layers value of permittivity ${\varepsilon}_r^{\prime }$ can be varied from 6 to 9 depending on content and type of nanocarbon filler. As for value of dielectric loss tangent, it is equal to 0.003 for epoxy layers and increases from 0.003 to 0.3 in composite layer with increase of filler content. Also, quasiperiodic PS4 (LH)^5^ structure was made, with defect layer (M) in the middle (magnetic composite layer BaM/L285 with BaM content of 30 wt.%; thickness of M-layer was 0.5 mm).

During the investigation of EMR shielding (frequency ranges of 26–37.5 GHz and 37.5–54 GHz) of photonic structures, the basic parameters, that were measured are the standing wave ratio by voltage (SWR), related to EMR reflection index *r* = (SWR − 1)/(SWR + 1) and transmission index *t*, which defines full attenuation of EMR during shield transition due to EMR reflection processes on layer boundaries and EMR absorption inside the shield. The samples of composite materials with cross section of 7.2 × 3.4 mm^2^ (or 2.6 × 5.2 mm^2^) were placed in cavity of rectangular waveguide and completely fill its cross section. The total thickness of PC samples was ~30 mm. Measurements were performed with scalar analyzers SHF P2-65 and P2-67.

## Results and Discussion

The main idea of the investigation of EMR transmission SE_T_(*f*) spectra for photonic structures is the determination of the influence on the position, width and depth of PBG of various parameters of PC, such as: (1) permittivity value of layers, (2) layer thickness, (3) the EMR absorption in H-layers with high real part of permittivity, and (4) the presence of defect layer, which has different permittivity or is magnetic.

It is expected that by arranging the composite layers (H) with filler (high permittivity) and the ones without it periodically, the PBG appears in EMR transmission spectrum [1, 2]. To implement such structures, it is necessary that these two types of material have a difference in permittivity (or refraction index) that is independent on frequency in investigated frequency range, and for pattern sharpness, they also must have low absorption index (close to zero).

Because we investigate microwave range, the layer thickness should be relatively high (~5–10 mm). To reduce it and the required number of layers in periodic structure, we need higher difference of real part of permittivity between layers of type L and type H while maintaining the imaginary part, which is responsible for absorption, minimal in both types.

As it was mentioned above, in epoxy composites filled with nanocarbon particles (graphite nanoplatelets or carbon nanotubes), we have observed the increase of the real part of dielectric permittivity ${\varepsilon}_r^{\prime }$ with increase of filler content. We also observed the increase of dielectric loss tangent in these composites, but the value of tan*δ* is comparatively low. Table 2 presents the data on ${\varepsilon}_r^{\prime }$, tan*δ* for some CMs based on the various types of epoxy resins—ED20 and SEDM2— which had been investigated earlier in our previous papers [32, 42].

**Table 2:** Electrical conductivity *σ*_*dc*_, dielectric permittivity ${\varepsilon}_r^{\prime }$, and dielectric loss tangent tan*δ* for the epoxy CMs versus carbon fillers content *C*

*C*, wt.%	*σ*_*dc*_ (293 К), S/m	*f*, GHz	${\varepsilon}_r^{\prime }$,	tan*δ*	Ref.
GNP/epoxy ED20					
1	1.0 · 10^−11^	27	4.9	0.017	[32, 43]
2	1.7 · 10^−11^		6.1	0.036	
5	1. 8 · 10^−9^		15	0.060	
10	0.12		52	0.175	
CNT(II)/epoxy SEDM2					
1	1.7 · 10^−10^	27	4.66	0.177	[43]
2	1.4 · 10^−10^		7.45	0.235	
3	3.8 · 10^−7^		8.58	0.260	
5	1.3 · 10^−4^		13	0.358	
10	1.7 · 10^−1^		22.75	0.730	
Epoxy resin L285					
–	8.0 · 10^−12^	27	2.9	0.003	This work
CNT/L285					
2	3.2 · 10^−5^	27	6	0.10	This work
		46	5.6	0.24	
GNP/L285					
5	2.8 · 10^−4^	27	8.9	0.06	This work
		46	8.4	0.11	
BaM/L285					
30	~1 · 10^−10^	27	3.3^a^	0.024^a^	This work
		37	3.4^a^	0.028^a^	

^a^ ${\varepsilon}_r^{\prime }$ and tan*δ* were determined at the assumption that ${\mu}_{\mathrm{r}}^{\prime}\approx 1$

As we can see from the Table 2, the values of ${\varepsilon}_r^{\prime }$ and tan*δ* depend not only on the type of carbon filler and its content, but also on the type of epoxy resin (its viscosity) and conditions of composite preparation, which significantly affect the filler dispersion (de-agglomeration) in CMs. For example, the composites based on epoxy resin ED20 filled with GNPs showed the larger values of dielectric permittivity ${\varepsilon}_r^{\prime }$ and the lower values of tan*δ* compared with epoxy composites filled with carbon nanotubes. In addition, the special fabrication method for such composite, when the composite mixture GNPs/epoxy was subjected to long ultrasonic dispersing (during 20 h) [43], promoted the covering of GNP particles with epoxy layer that prevents the agglomeration of GNP particles and lead to the increase of the dielectric permittivity of CMs and to the increase of the percolation threshold. Sometimes, the observed lower value of dielectric permittivity of CNT-filled composites compared to GNP-filled CMs is related to high agglomeration of CNTs due to its large entanglement. So, varying the types of epoxy resin, carbon filler content, and method of composite fabrication, we can achieve the optimal values of dielectric permittivity ${\varepsilon}_r^{\prime }$ and dielectric loss tangent tan*δ*. As a result, for fabrication of photonic structures, the composite mixture 2 wt.%CNT/epoxy L285 and 5 wt.%GNPs/epoxy L285 were used as H-layers with high dielectric permittivity. The use of epoxy resin L285 with low viscosity compared with epoxy resins ED20 and SEDM2 excludes the use of acetone during the composite preparation.

Firstly, we have modeled the transmission spectra for PC with epoxy and epoxy-filled layers with known material parameters ${\varepsilon}_{r1}^{\prime }$ and ${\varepsilon}_{r2}^{\prime }$. PS structure consisted of ten layers (five periods, (LH)^5^) and placed in rectangular waveguide. For simulating the EMR transmission spectra for PC, the transmission line (TL) method is being used, when the reflection coefficient at the surface of the first layer is obtained by starting the calculations from the last layer using the impedance matching concept. This calculating method has been implemented in Wolfram Mathematica and C++ code.

**Fig. 1:**
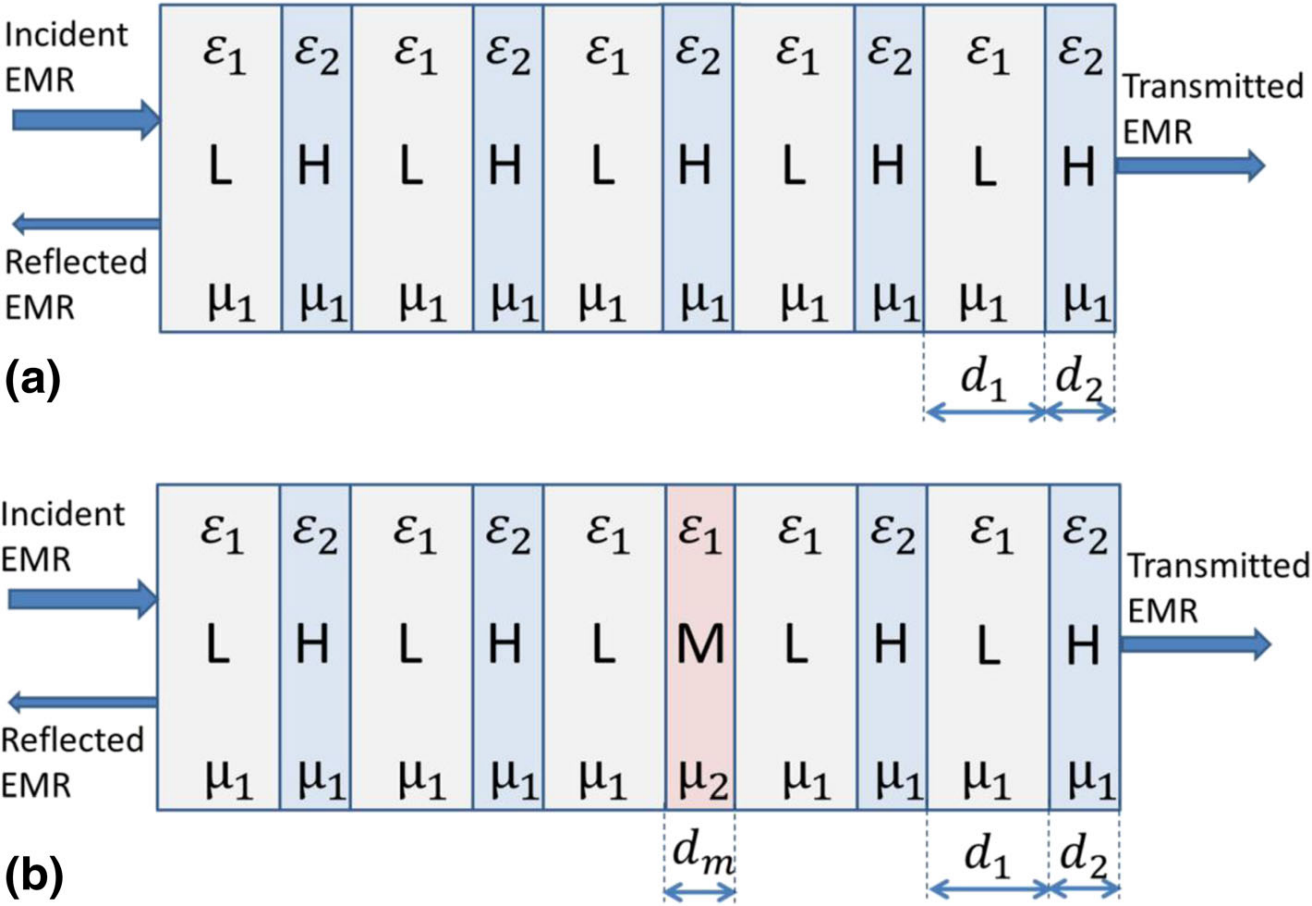
Model representation of PC transmission by EMR. **a** Ideal PC structure. **b** PC structure with one defect magnetic layer

Wave transmission in PC can be presented as shown in Fig. 1.

Thus, we have a set of layers to which we can apply TL method and determine input and output impedances for each layer.

For the measurements in rectangular waveguide, the frequency dependencies of EMR transmission *SE*_*T*_(*f*) and reflection *RL*(*f*) indexes within TL method are described by the following relations [44]:

1 $$ \mathrm{S}{\mathrm{E}}_{\mathrm{T}}=20 \log \left({\displaystyle \prod_{j=1}^n\left|\frac{\left({X}_{\mathrm{j}}+{Z}_{\mathrm{j}}\right)}{\left({X}_{\mathrm{j}}+{Z}_{\mathrm{j}+1}\right)}\cdot \exp \left(-{\gamma}_{\mathrm{j}}{d}_{\mathrm{j}}\right)\right|}\right), $$

2 $$ \mathrm{R}\mathrm{L}=20 \log \left|\frac{\left({X}_{\mathrm{n}}-{Z}_{\mathrm{n}+1}\right)}{\left({X}_{\mathrm{n}}+{Z}_{\mathrm{n}+1}\right)}\right|, $$

where *Z*_j_(*X*_j_)—input (output) impedance for *j*th layer, determined as following:

3 $$ {Z}_{\mathrm{j}}=\frac{Z_0}{\sqrt{\varepsilon_{\mathrm{rj}}^{\ast }{\mu}_{\mathrm{rj}}^{\ast }-{\left(\frac{\lambda}{2 a}\right)}^2}}, $$

4 $$ {X}_{\mathrm{j}}=\frac{Z_j\left({X}_{\mathrm{j}-1}+{Z}_{\mathrm{j}}\cdot \tanh \left({\gamma}_{\mathrm{j}}\cdot {d}_{\mathrm{j}}\right)\right)}{\left({Z}_{\mathrm{j}}+{X}_{\mathrm{j}-1}\cdot \tanh \left({\gamma}_{\mathrm{j}}\cdot {d}_{\mathrm{j}}\right)\right)}, $$

5 $$ {\gamma}_{\mathrm{j}}= i\cdot {k}_{\mathrm{zj}},{k}_{\mathrm{zj}}={k}_0\cdot \sqrt{\varepsilon_{\mathrm{rj}}^{\ast }{\mu}_{\mathrm{rj}}^{\ast }-{\left(\frac{\lambda}{2 a}\right)}^2}, $$

where ${Z}_0=\sqrt{\mu_0/{\varepsilon}_0}=377\;\varOmega$; *k*_0_ = 2*π*/*λ* is the wave vector in free space, *λ* = *C*_0_/*f*; *λ* and *f* are the wavelength and the frequency; ${\varepsilon}_{\mathrm{r}}^{*}={\varepsilon}_{\mathrm{r}}^{\prime }- i{\varepsilon}_{\mathrm{r}}^{{\prime\prime} }={\varepsilon}^{\prime}\left(1- i\cdot \tan {\delta}_{\upvarepsilon}\right)$ and ${\mu}_{\mathrm{r}}^{*}={\mu}_{\mathrm{r}}^{\prime }- i{\mu}_{\mathrm{r}}^{{\prime\prime} }={\mu}_{\mathrm{r}}^{\prime}\left(1- i\cdot \tan {\delta}_{\upmu}\right)$ are the relative complex permittivity and the relative complex permeability of medium, respectively; *d*_j_ is layer thickness; *a =* 7.2 or 5.2 mm —the inside width of rectangular waveguide.

### Modeling of EMR Transmission Index for 1D Photonic Structures in Rectangular Waveguide in Frequency Ranges 26–37.5 GHz and 37.5–54 GHz

Output parameters of L-layers with low permittivity, were chosen as follows: ${\varepsilon}_{r1}^{\prime }=2.9$; tan*δ*_ε1_ = 0.003; *d*_1_ = 5 mm. Thickness of L-layers in 5 mm was chosen as such so that optical thickness $\sqrt{\varepsilon_{\mathrm{r}1}^{\prime }}\cdot {d}_1\approx 3\cdot {\lambda}_0/4$, so in frequency range 3–54 GHz, we will observe three PBGs. Increase of the number of PBGs, and their narrowing allows us to observe full band gaps in relatively narrow frequency ranges 26–37.5 GHz and 37.5–54 GHz, accordingly.

Values of H-layers parameters with relatively high permittivity were varied in certain ranges, in order to define their influence on PBG characteristics in PS (LH)^5^. Calculations of EMR transmission spectra were performed for such variations of H-layer parameters:

1. $$ {\varepsilon}_{\mathrm{r}2}^{\prime } $$ =5, 5.6, 7, 9, 11, 20; tan*δ*_*ε*2_ =0.003; *d*_2_ =1 mm;
2. ${\varepsilon}_{\mathrm{r}2}^{\prime }$ =6; tan*δ*_*ε*2_ =0.003; *d*_2_ =0.6, 0.8, 1, 1.2, and 1.4 mm;
3. ${\varepsilon}_{\mathrm{r}2}^{\prime }$ =6; tan*δ*_*ε*2_ =0.1; *d*_2_ =0.6, 0.8, 1, 1.2, and 1.4 mm.

**Fig. 2:**
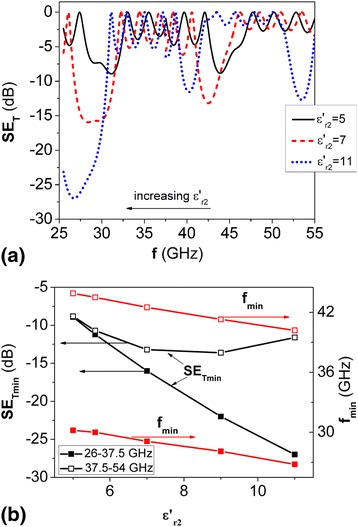
Calculated EMR transmission spectra (**a**) for PS (LH)^5^ at various values of permittivity ${\varepsilon}_{r2}^{\prime }$ of H-layers and dependencies of position and depth of PBG on permittivity value ${\varepsilon}_{r2}^{\prime }$ of H-layers (**b**); ${\varepsilon}_{r1}^{\prime } = 2.9$; *d*_1_ = 5 mm; *d*_2_ = 1mm; tan*δ*_*ε*2_ = 0.003

Figure 2a depicts the frequency dependencies of EMR transmission index, simulated for various values of permittivity of H type layers.

Analysis of the obtained data, as shown in Fig. 2b, indicated that during the increase of permittivity the shift of PBGs occurs to lower frequencies and for PS with ${\varepsilon}_{\mathrm{r}2}^{\prime }=11$ we observed three PBGs. As for band gap depth, it increased with the increase of permittivity of H type layers, especially for frequency range 26–37.5 GHz.

**Fig. 3:**
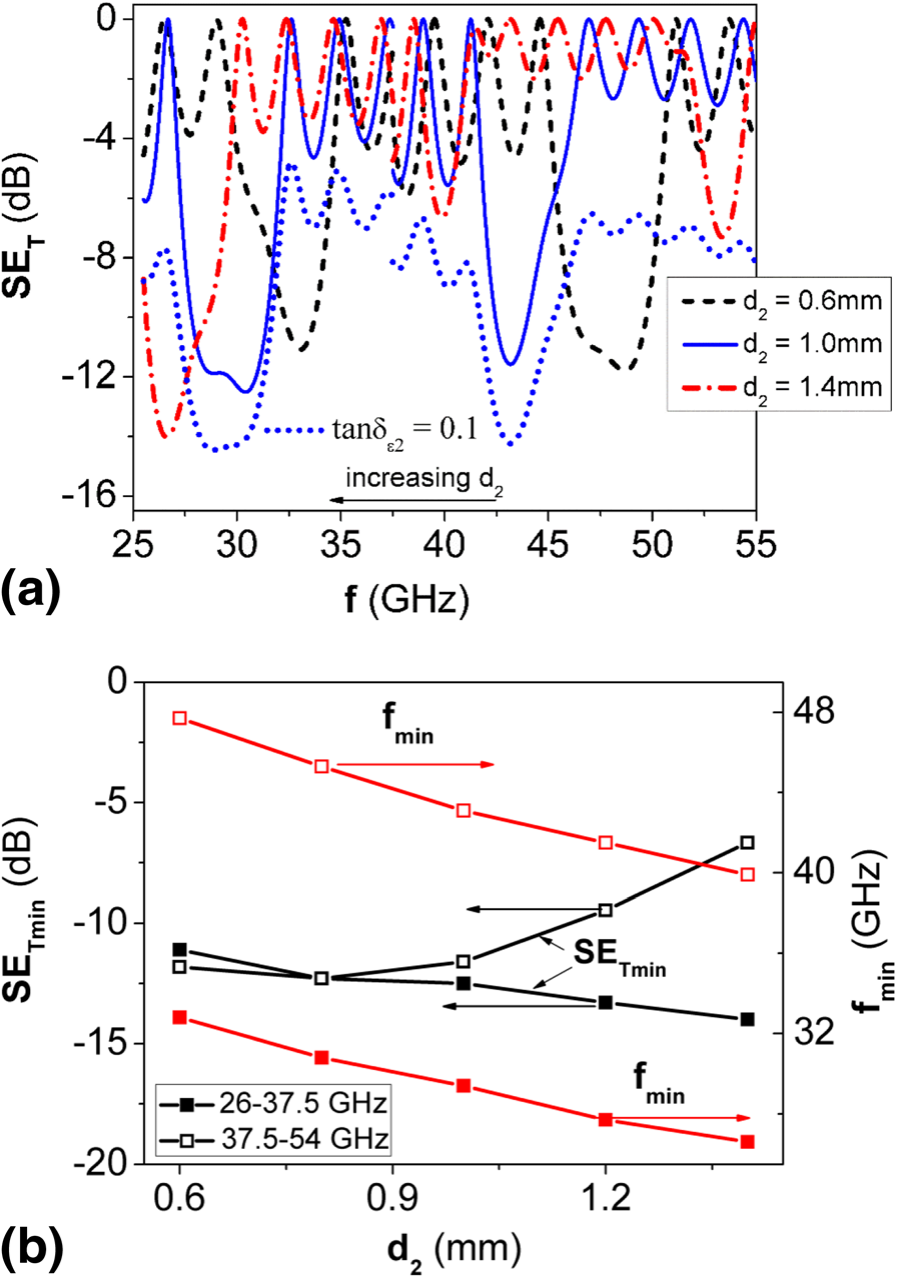
Calculated EMR transmission spectra (**a**) for 1D PS (LH)^5^ at various values of thickness *d*_2_ of H-layers and dependencies of position and depth of PBG on thickness *d*_2_ of H-layers (**b**); ${\varepsilon}_{r1}^{\prime } = 2.9$; *d*_1_ = 5mm; ${\varepsilon}_{r2}^{\prime } = 6$; tan*δ*_*ε*2_ = 0.003. *Short-dotted curve* on Fig. 3a corresponds to case, when *d*_2_ = 1 mm and tan*δ*_*ε*2_ = 0.1

Figure 3a presents the frequency dependencies of EMR transmission index for PS, calculated at the value of permittivity of H type layers ${\varepsilon}_{\mathrm{r}2}^{\prime }=6$ and tan*δ*_*ε*2_ = 0.003 for various thicknesses of H-layers. Figure 3b presents the variations of PBG position *f*_min_ and depth *SE*_T min_, depending on layer thickness *d*_2_.

As it is seen from the figures, the increase of H type layer thickness causes the shift of PBG to lower frequencies, marginal narrowing of band gaps and for PS with *d*_2_ = 1.4 mm_,_ we observed three PBGs. The depth of the first PBG (in frequency range 26–37.5 GHz) increases with the increase of layer thickness *d*_2_ while depth of second PBG (for frequency range 37.5–54 GHz) first, somewhat, increased and then significantly decreased.

Calculation of spectra of EMR transmission index for PS with H type layers with non-zero EMR absorption index (tan*δ*_*ε*2_ = 0.1) showed the shift of spectrum down by *ΔSE*_T_ = 5 − 6 dB and, accordingly, increase of the *SE*_*T* min_ value, though the pattern of changes of values *f*_min_, *Δf* and *SE*_T min_ are similar to case tan*δ*_*ε*2_ = 0.003 (Fig. 3a).

### Comparative Analysis of Simulated and Experimental Data on EMR Transmission Index for 1D Photonic Structures (LH)^5^ in Rectangular Waveguide in Frequency Ranges 26–37.5 GHz and 37.5–54 GHz

**Fig. 4:**
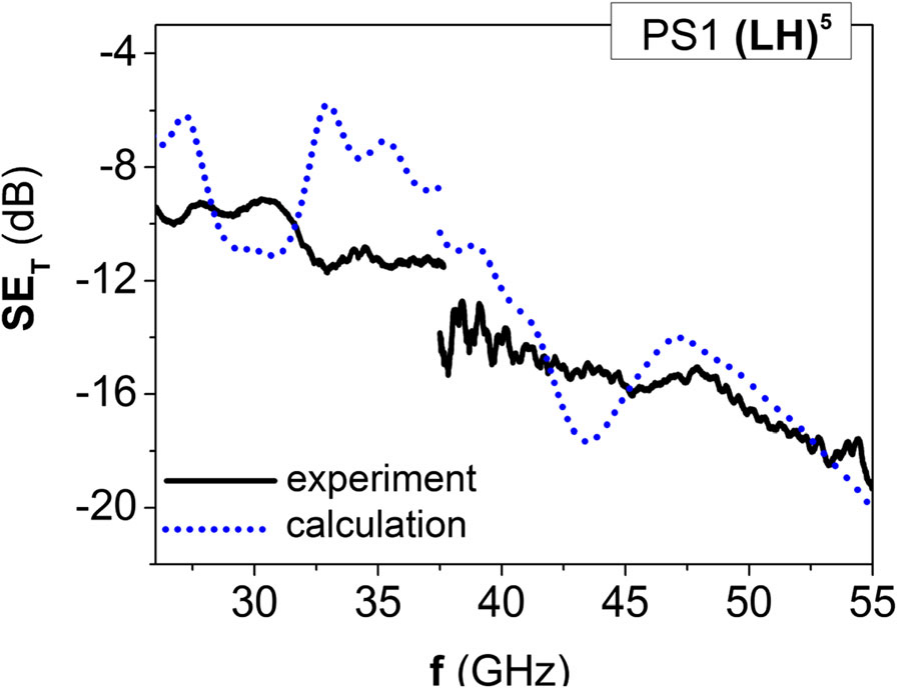
EMR transmission spectra for 1D PS1 (LH)^5^, that consists of five epoxy L285 layers, which alter with composite layers 2 wt.%CNT/L285: *solid lines* correspond to experiment, *dotted lines* represent calculation at layers parameters listed in Tables 1 and 2 and tan*δ*_*ε*2_ = − 0.098 + 0.0073*f*

Figure 4 represents the experimental frequency dependencies of EMR transmission index for PS1 (LH)^5^, which consists of five layers of L285, which alter sequentially with composite layers 2 wt.%CNT/L285. Data on layers thickness of PS1 are presented in Table 1, composite layer permittivity ${\varepsilon}_{\mathrm{r}2}^{\prime }$ and dielectric loss tangent tan*δ*_*ε*2_ were determined by short-circuited line method [31] and are presented in Table 2.

Using the layer parameters of PS1, frequency dependence of EMR transmission index has been calculated and compared with measured EMR transmission spectra for PS1. At first iteration, we consider that ${\varepsilon}_{\mathrm{r}2}^{\prime }=6$ is not dependent on frequency in the range 26–37.5 GHz. Similarly, for the range 37.5–54 GHz ${\varepsilon}_{\mathrm{r}2}^{\prime }=5.6$ and is constant in this frequency range. The measured values of tan*δ*_*ε*2_ are 0.1 at the frequency 27 GHz and 0.24 at 46 GHz, and for simulation, the frequency dependence of dielectric loss was approximated by the relation tan*δ*_*ε*2_ = − 0.098 + 0.0073*f*. The calculated EMR transmission spectra for PS1 with layer parameters, presented above, are shown in Fig. 4 by dashed lines. As it is seen from the presented data, the sharp PBGs for prepared PS1 on experimental EMR transmission spectra are not observed, and they differ considerably from simulated EMR transmission spectra. Such differences between experimental and modeling spectra can be caused by several reasons:

1. Imperfection of PS layers, namely, layer thickness and filler content in H type layers, which affects permittivity considerably.
2. Small difference between permittivities of L and H type layers, which considerably reduces the depth of PBG.
3. EMR absorption in H-layers, which decreases the EMR transmission index in bands, should correspond to full transmission of EMR.

In order to increase the value of the permittivity of H-layers in PC and to reduce the effect of EMR absorption in these H-layers on the sharpness of photonic band gap in transmission spectra, we used epoxy layers filled with 5 wt.% of GNPs and decreased the thickness of composite H-layers.

**Fig. 5:**
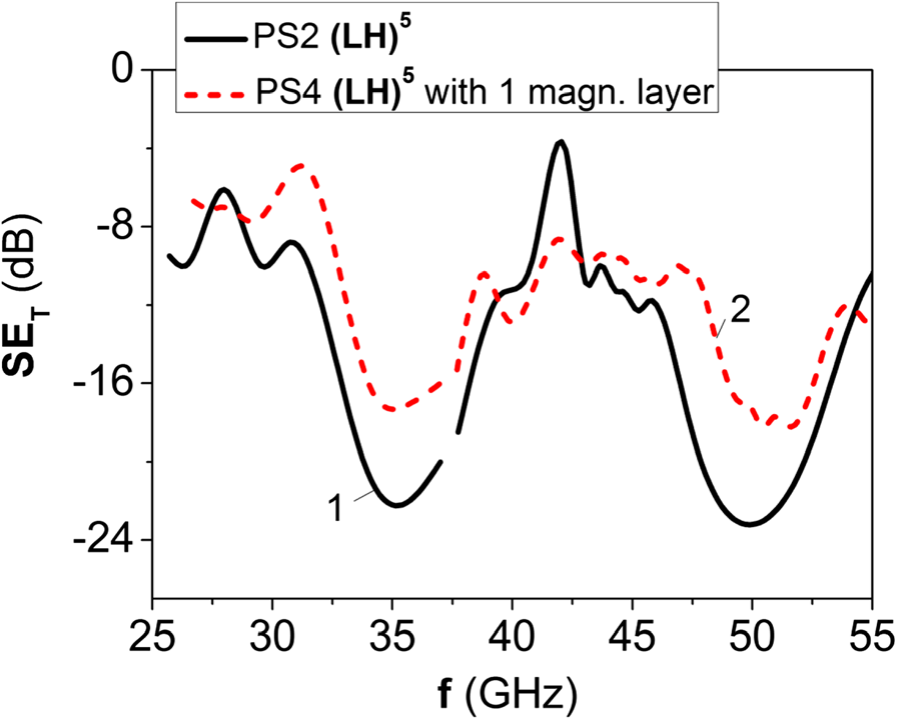
EMR transmission spectra for 1D PS2 (LH)^5^, that consists of five epoxy layers L285, which alter with composite layers 5 wt.%GNP/L285 (*curve 1*, *solid line*) and for PS4 (LH)^5^ with one magnetic M-layer (30 wt.%BaM/L285) instead of one 5 wt.%GNP/L285 H-layer (*curve 2*, *dashed line*)

Figure 5 presents the data on EMR transmission spectra for PS2 (LH)^5^, which consists of five layers of epoxy L285 and five composite layers 5 wt.%GNP/L285.

Thickness of pure epoxy layers was 4.8 mm, thickness of composite layers was 0.48 mm, the measured composite layer permittivity ${\varepsilon}_{\mathrm{r}2}^{\prime }$ was 8.9 at 27 GHz and slightly decreased down to 8.4 at 46 GHz (see Tables 1 and 2). The changes of measured dielectric loss tangent versus frequency were approximated by the relation tan*δ*_*ε*2_ = − 0.0198 + 0.003*f*. As it is seen from Fig. 5, the increase of permittivity ${\varepsilon}_{\mathrm{r}2}^{\prime }$ of composite layers up to ~ 9 and decrease of their thickness *d*_2_ (for decreasing of EMR absorption in composite layers) as compared with PS1 allow to observe more marked photonic band gaps in PS2. The insertion of defect layer in PS4 (magnetic composite layer 30 wt.%BaM/L285 with EMR absorption due to non-zero tan*δ*_*μ*2_) leads to some changes in the shape and depth of PBG, while the position of PBG is retained. As it is seen from Fig. 5, the passband inside of PBG is not observed for such parameters of magnetic layer as listed in Tables 1 and 2. Maybe, more significant changes of PBG in photonic structures with magnetic layer should be observed under the action of external magnetic field that leads to anisotropic magnetic properties of M-layer. Further detailed research is needed to clarify these issues.

**Fig. 6:**
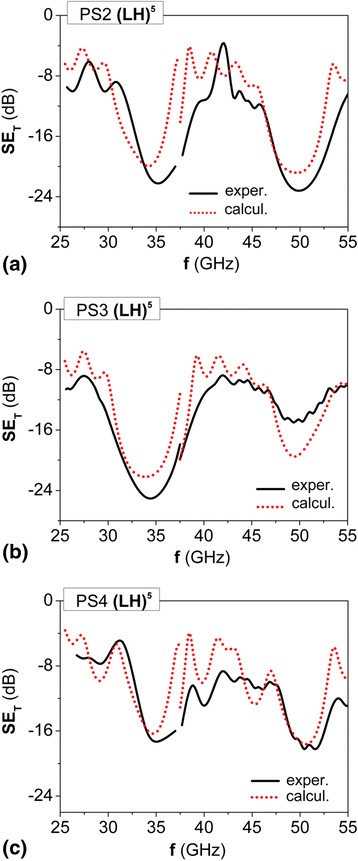
EMR transmission spectra for 1D PS2 (**a**) and PS3 (**b**) (LH)^5^ structures which consist of five epoxy layers L285, which alter with composite layers 5 wt.%GNP/L285 with various thickness of composite layers; (**c**) photonic structure PS4 with one magnetic M-layer; *solid lines*—experimental data, *dotted lines*—calculation at parameters listed in Tables 1 and 2

Figure 6a, b present the experimental data on transmission index for PS2 and PS3, depending on the thickness of L- and H-layers.

As it is seen, the increase of H-layers thickness *d*_2_ in PS3 sample compared with PS2 sample leads to shift of PBG in the range of lower frequencies and lower values of PBG depth *SE*_T min_. Such behavior of *SE*_T_(*f*) spectra is explained by higher absorption of EMR due to increased thickness of composite H-layers with non-zero tan*δ*_2_. The modeling of EMR transmission spectra for PS2, PS3, and PS4 structures with layers parameters listed in Tables 1 and 2 (see Fig. 6, dotted lines) showed good agreement with the experimental data.

## Conclusions

The modeling of EMR transmission spectra for 1D periodic photonic structure (LH)^5^, placed in rectangular waveguide (frequency range 26–37.5 GHz and 37.5–54 GHz) were carried out. The performed simulation allowed us to determine the influence of parameters of layers, which compose the photonic structure, on the position, number, width, and depth of photonic band gaps (PBGs).

It was found that the depth *SE*_T min_ of PBG increases and shift of PBGs (*f*_min_) occurs to lower frequencies with increase of the composite layers permittivity, and the value of ${\varepsilon}_2^{\prime }/{\varepsilon}_1^{\prime }$ ratio. It was shown, that at optical thickness of photonic crystal layers, that is a multiple of *λ*_0_/4, the few PBGs were observed, and the number of PBGs is equal to multiple number *n*. Also the width of PBGs is reduced proportionally to their number.

The experimental investigation of EMR transmission spectra *SE*_T_(*f*) in the frequency ranges 26–37.5GHz and 37.5–54 GHz for fabricated 1D periodic PSs (LH)^5^, based on epoxy layers and composite layers, filled with nanocarbon particles (graphite nanoplatelets or carbon nanotubes) has shown the presence of few PBGs due to increased optical thickness of epoxy layers up to ~ 3 ⋅ *λ*_0_/4. It was also found that the EMR absorption in composite H-layers shifts *SE*_T_(*f*) spectra to the smaller values of EMR transmission index and reduces the depth of PBGs.

Based on comparative analysis of the experimental and calculated EMR transmission spectra for investigated photonic structures, it was concluded that for observing the clear marked PBGs in such PS it is necessary to provide a significant difference between permittivities of L- and H-layers, minimize EMR absorption in composite layers H (tan*δ*_*ε*2_ should be lower than 0.1), and ensure the layers of each type to be identical.

## Abbreviations

CNT: Carbon nanotube
EMR: Electromagnetic radiation
GNP: Graphite nanoplatelet
PBG: Photonic band gap
PC: Photonic crystal
PS: Photonic structure
*SE*_T_(*f*): EMR transmission spectrum
